# Supplementary figures and images for: Co-Targeting PD-1 and IL-33/ST2 Pathways for Enhanced Acquired Anti-Tumor Immunity in Breast Cancer
Source: Int J Mol Sci. 2025 Oct 1;26(19):9600. doi: 10.3390/ijms26199600 (PMC12525228; doi:10.3390/ijms26199600)

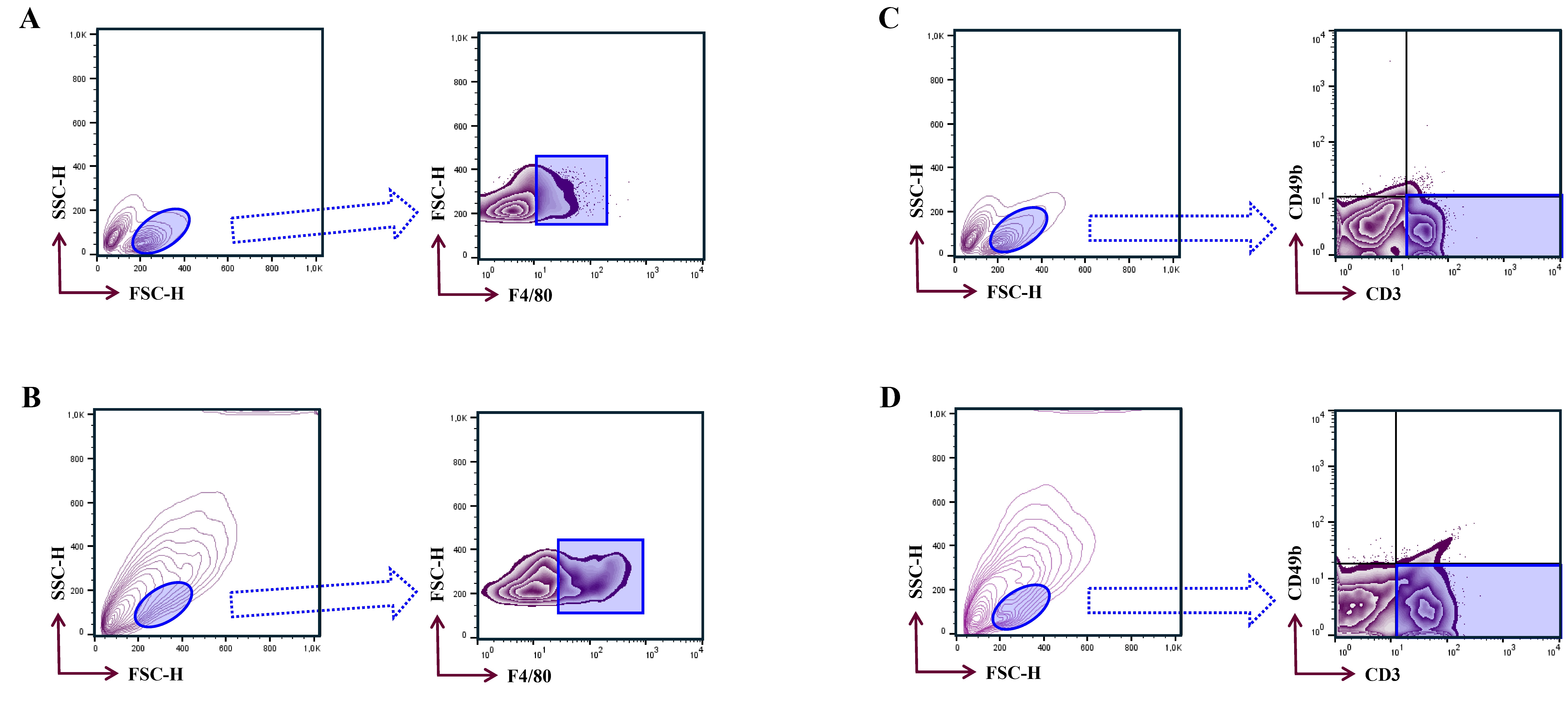

Supplement: Supplementary file 1 [file ijms-26-09600-s001.zip › ijms-3844154-supplementary/Figure S1/Figure S1.jpeg]
